# Silicon compounds in carbon-11 radiochemistry: present use and future perspectives

**DOI:** 10.1039/d1ob01202a

**Published:** 2021-07-20

**Authors:** Federico Luzi, Antony D. Gee, Salvatore Bongarzone

**Affiliations:** School of Biomedical Engineering and Imaging Sciences, 4th floor Lambeth Wing, St Thomas’ Hospital, King's College London London SE1 7EH UK antony.gee@kcl.ac.uk salvatore.bongarzone@kcl.ac.uk

## Abstract

Positron emission tomography (PET) is a powerful functional imaging technique that requires the use of positron emitting nuclides. Carbon-11 (^11^C) radionuclide has several advantages related to the ubiquity of carbon atoms in biomolecules and the conservation of pharmacological properties of the molecule upon isotopic exchange of carbon-12 with carbon-11. However, due to the short half-life of ^11^C (20.4 minutes) and the low scale with which it is produced by the cyclotron (sub-nanomolar concentrations), quick, robust and chemospecific radiolabelling strategies are required to minimise activity loss during incorporation of the ^11^C nuclide into the final product. To address some of the constraints of working with ^11^C, the use of silicon-based chemistry for ^11^C-labelling was proposed as a rapid and effective route for radiopharmaceutical production due to the broad applicability and high efficiency showed in organic chemistry. In the past years several organic chemistry methodologies have been successfully applied to ^11^C-chemistry. In this short review, we examine silicon-based ^11^C-chemistry, with a particular emphasis on the radiotracers that have been successfully produced and potential improvements to further expand the applicability of silicon in radiochemistry.

## Abstract 2

The use of silicon-based reagents and precursors for carbon-11 labelling has shown wide applicability and robustness with short reaction times using mild conditions. In this review, recent advances and future perspectives are examined.

## Graphical abstract

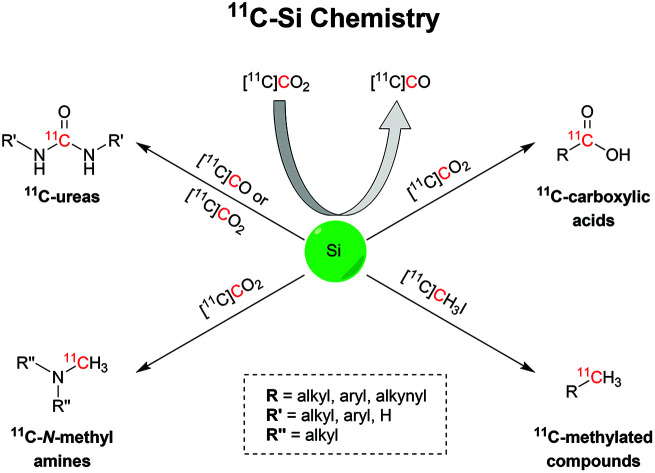

## 1. Introduction

Positron emission tomography (PET) is a powerful functional imaging technique that allows the *in vivo* detection of normo- and patho-physiological changes in humans by using molecules radiolabelled with positron (β^+^) emitting nuclides (radiotracers).^1^ To achieve radiopharmaceutical targeting, radiotracers are often derived from biologically-active compounds with a known pharmacological profile, possessing high selectivity for a molecular target or physiological process.^2^ The inclusion of a positron-emitting nuclide in the molecule of interest enables the *in vivo* visualization of the molecules biodistribution and kinetics metabolism.^2,3^ Of all the available PET nuclides, carbon-11 (^11^C) is of particular interest due to the ubiquity of carbon atoms in biomolecules and because isotopic substitution of carbon-12 for carbon-11 preserves the biological properties of the non-radioactive isotope.^2,4,5^ However, due to the rapid radioactive decay of carbon-11 (radioactive half-life *t*_1/2_ = 20.4 minutes), the radiosynthesis, purification, formulation and quality control of carbon-11 radiopharmaceuticals must be accomplished in short times (the whole process should not exceed 60 minutes), hence quick and robust chemistry are needed to avoid substantial activity loss.^2,6^ The sub-nanomolar scale with which the radioisotope is produced from the cyclotron also represents a burden when performing ^11^C-labelling, with the non-radioactive reactants being in large stoichiometric excess. Each minor impurity in the solvents and the reagents may generate side-products or degradation of reagents resulting in unwanted intermediates, so a high degree of chemospecificity is required, as well.^2^

To meet criteria suitable for ^11^C-labelling, silicon-containing compounds have received increased interest in the field. Organosilicon compounds have already demonstrated wide applicability in traditional organic chemistry enabling the development of well-known methodologies such as the Hiyama cross-coupling reactions for the formation of carbon–carbon bonds between silylated compounds and aryl halides,^7,8^ tosylates,^9^ mesylates,^10^ sulfinates^11^ and phosphates^12^*via* palladium catalysis and fluoride or base activation.^7–12^ Moreover, silyl compounds act as effective protecting groups due to the large number of functional groups that can be protected (*e.g.* alcohols, alkynes, amines, carboxylic acids…) and the ease of the protecting/deprotecting steps.^13,14^ Another interesting application involves the use of hydrosilicon compounds, possessing one or more Si–H bonds, for the reduction of carbon dioxide (CO_2_) to more reactive species.^15–18^ In the hydrosilylation reaction, CO_2_ is used as a building block for the synthesis of a variety of functional groups such as formamides,^15^ methylamines,^15,16^ aldehydes^17^ and aminals.^18^ Moreover, organosilicates have shown to be optimal substrates for the electrophilic fluorination of aryl and alkenyl substrates under mild conditions (*e.g.* room temperature, 18 hours) whilst having regio- and enantio-selectivity on the final product.^19^ Besides the large number of reactions available, silicon chemistry is cost-effective (the silylated reagents are easily synthesized or commercially available)^20^ and eco-friendly (organosilicon compounds are ultimately catabolised into silica gel in the environment).^7,8^

Given the high versatility and ease of handling of organosilane compounds, several methodologies have been successfully translated into carbon-11 and fluorine-18 chemistry in the past years. In particular, silicon-based compounds were applied in the production of a variety of fluorine-18 labelled small molecules and peptides as prosthetic groups, where the radionuclide was attached on *via* isotopic exchange (obtaining silicon-fluoride-acceptors – SiFAs),^21^ or as substrates for electrophilic fluorination.^19^ The application of silicon in fluorine-18 chemistry was recently reviewed by Bernard-Gauthier *et al.* and Tredwell *et al.*^19,21^

**Scheme 1:**
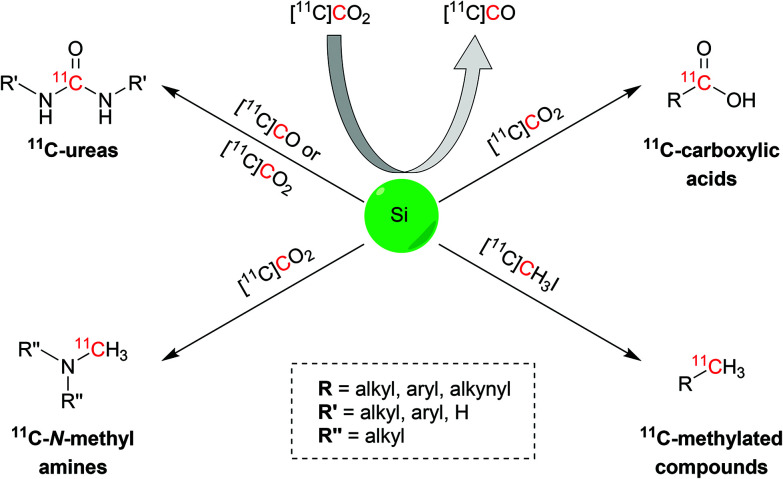
Schematic representation of the ^11^C functional groups and synthons obtainable *via* silicon chemistry.

Herein we report the latest advances in the ^11^C-chemistry field based on silicon starting materials or reagents (Scheme 1). This review will initially disclose the atomic properties of silicon to then discuss the main uses of organosilane compounds in carbon-11 chemistry: (i) conversion from [^11^C]CO_2_ to [^11^C]CO; (ii) trapping and activating agents for [^11^C]CO_2_ and (iii) precursors of the target radiopharmaceutical. This review will also report the biologically-active molecules that were successfully radiolabelled with the discussed reactions.

## 2. Characteristics and application of silicon in ^11^C-chemistry

### 2.1. Atomic properties of silicon

The versatility of silicon in organic chemistry is explainable by examining the strength of the bonds that this atom creates with other elements. The strength of Si–H and Si–C bonds (90 and 85 kcal mol^−1^, respectively) in organosilicates is indeed slightly lower than C–H and C–C bonds (104 and 88 kcal mol^−1^, respectively) in hydrocarbons, making silicon bonds more easily cleavable and silicates a favoured leaving group.^22–25^ The lower electronegativity of silicon would also generate a partial negative charge on carbon upon Si–C bond cleavage, favouring a higher reactivity on that carbon towards electrophiles.^22–24^ The higher electropositivity of silicon also favours the formation of hydride ions upon Si–H bond cleavage, making organosilicon compounds functional reducing agents.^25^ The high fluoro- and oxo-philicity of silicon also provokes the rearrangement or the cleavage of existing Si–C, Si–H and Si–Si bonds upon addition of fluorinated or oxygenated nucleophiles resulting in the formation of Si–F or Si–O bonds that enhances the nucleophilicity of the atom it was initially bound to, facilitating the reaction with electophiles.^22–24^ The involvement of d-orbitals further expands the applicability of silicon in radiochemistry, allowing the formation of penta- and hexa-coordinate compounds (*e.g.* the formation transition metal silylene complexes)^26^ which opens to more reaction pathways.^26,27^

### 2.2. Use of silyl compounds as [^11^C]CO_2_-to-[^11^C]CO converting agents

Carbon monoxide-releasing molecules (CORMs) have been exploited in the past years with either therapeutic or synthetic purposes.^28–30^ Within these compounds, silacarboxylic acids can produce CO upon exposure to high temperature or reaction with nucleophiles (*e.g.* fluoride anions, bases, water) following the 1,2-Brook rearrangement^28–33^ and can be readily synthesized by the reaction of the relative silyl lithium derivative with CO_2_.^29,30^ The produced CO was then employed in carbonylative coupling of aryl iodides and amines to yield amides, esters and α,β-unsaturated ketones.^29,30^

Considering these advantageous characteristics, silacarboxylic acids were examined as potential [^11^C]CO_2_-to-[^11^C]CO converting agents as an alternative strategy to gas-phase reduction (reduction over thin molybdenum wires at 850 °C),^34^ photo-chemical (a ruthenium/cobalt solution irradiated by visible light)^35^ and electro-chemical methods (using transition-metal triflates as cathodes).^36^

**Scheme 2:**
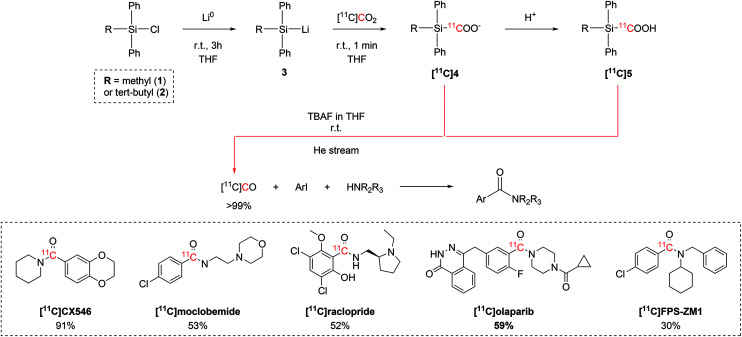
[^11^C]CO_2_-to-[^11^C]CO conversion *via*^11^C-silanecarboxylate derivatives, ^11^C-carbonylation of aryl iodides and biologically-active molecules successfully synthesized with the produced [^11^C]CO.^37–39^

Initial efforts focused on identifying the organosilyl chloride with the highest reactivity towards [^11^C]CO_2_. A variety of tri-substituted silanes were tested, with chloro(methyl)diphenylsilane (**1**) and chloro(*tert*-butyl)diphenylsilane (**2**) showing the overall best trapping efficiency (TE >95%) after reacting with metallic lithium to yield the corresponding lithium silane (**3**, Scheme 2). The reaction with [^11^C]CO_2_ forms the relative ^11^C-silacarboxylate ([^11^C]**4**) which could be protonated by adding HCl to obtain ([^11^C]**5**). Both [^11^C]**4** and [^11^C]**5** are able to release [^11^C]CO after tetrabutylammonium fluoride (TBAF) addition.^37–39^ A quantitative release of [^11^C]CO was achieved by adjusting reaction variables such as the amount of the starting chlorosilane, the temperature, equivalents of TBAF, the reaction time, the solvent.^37–39^ The [^11^C]CO produced with these optimised conditions was then employed in the palladium-catalysed ^11^C-carbonylation of several molecules of interest (Scheme 2), including the selective AMPA ligand [^11^C]CX546, the monoamine oxidase A (MAO-A) inhibitor [^11^C]moclobemide, the dopamine D2 selective antagonist [^11^C]raclopride, [^11^C]olaparib for cancer imaging and the receptor for advanced glycation end-products (RAGE) radiotracer [^11^C]FPS-ZM1 in radiochemical yields (RCYs) ranging between 29% and 91% (estimated by radioHPLC).^37–39^ Follow-up studies on the ^11^C-silacarboxylate production of [^11^C]CO and subsequent ^11^C-carbonylation showed that the whole process can be fully automated using a commercially available radiochemistry synthesis module.^38^ With an initial delivery of 10 GBq of [^11^C]CO_2_, the radiotracer [^11^C]FPS-ZM1 (Scheme 2) was achieved with a RCY of 34% (isolated, decay-corrected to end of [^11^C]CO_2_ delivery (EOD)) and molar activity (*A*_m_) 28 GBq per μmol within 25 minutes from end of bombardment (EOB).^38^

**Scheme 3:**
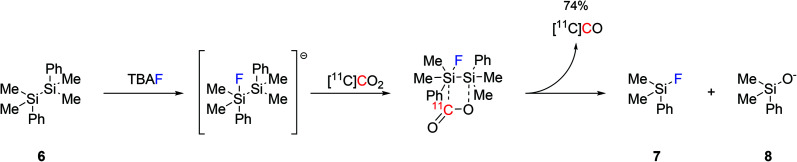
Proposed mechanism for the fluoride-promoted [^11^C]CO production from disilane species.^40^

Another approach to produce [^11^C]CO was reported by reacting disilanes with [^11^C]CO_2_. Four different disilane species were tested for their ability of trapping [^11^C]CO_2_ and converting it into [^11^C]CO, with 1,2-diphenyl-1,1,2,2-tetramethyldisilane ((Me_2_PhSi)_2_, **6**, Scheme 3) being the most effective.^40^ With the aid of catalytic amounts of TBAF (0.1 equiv.) as a fluoride source, the production of [^11^C]CO reached 74% using mild reaction conditions and within 3 minutes from EOB.^40^ The reaction mechanism is believed to proceed by the initial formation of a pentavalent fluorosilyl anion which rapidly interacts with [^11^C]CO_2_ forming an unstable intermediate that spontaneously rearranges into a silylfluoride derivative (**7**), a silyloxide derivative (**8**) and [^11^C]CO (Scheme 3). As a proof-of-concept, the applicability of the produced [^11^C]CO in ^11^C-carbonylation reactions was tested *via* the radiosynthesis of [^11^C]benzylbenzamide which was obtained with a good RCY (74%, estimated by radioHPLC).^40^

### 2.3. Reductive functionalisation of [^11^C]CO_2_ using organosilanes

**Scheme 4:**
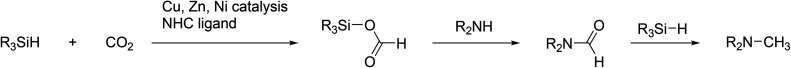
Hydrosilylation of CO_2_ forming a silyl formate. The reaction of an amine with a silyl formate produces a formamide which can be reduced by an excess of hydrosilane to yield an *N*-methylamine.^16^

The high reactivity that organosilanes have towards CO_2_ enabled other interesting applications, such as the conversion of the cyclotron-produced [^11^C]CO_2_ to a ^11^C-*N*-methyl group without the need of using [^11^C]CH_3_I. This approach was vastly exploited in synthetic chemistry to use CO_2_ as a C_1_ building block in hydrosilylation reactions of CO_2_.^16,41,42^ The gas is initially trapped by the organosilicon compound in the form of silyl formate (Scheme 4)^41^ with the aid of transition-metal catalysis (*e.g.* Cu, Zn, Ni) and bulky ligands (*e.g.* σ-donor *N*-heterocyclic carbenes, NHCs).^16,41,42^ Then, the carbonyl group reacts with the target amino compound to form formamides (Scheme 4).^42^ The presence of an excess of silane provokes the reduction of the formamide to *N*-methylamine (Scheme 4).^16^

**Scheme 5:**
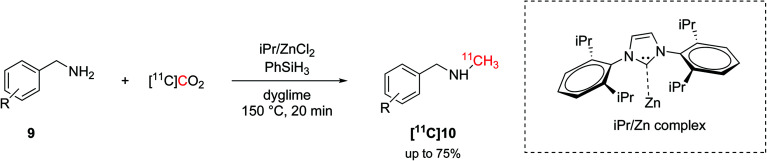
Hydrosilylation of [^11^C]CO_2_ and application on the ^11^C-*N*-methylation.^43^

The hydrosilylation of CO_2_ was later translated into carbon-11 chemistry by Liger *et al.*^43^ Similarly to the aforementioned non-radioactive reactions,^16^ this methodology exploited a NHC (1,3-bis(2,6-diisopropylphenyl)-1,3-dihydro-2*H*-imidazol-2-ylidene, iPr, Scheme 5) as ligand, zinc catalysis and high temperature (150 °C) to yield ^11^C-*N*-methylamines within 20 minutes from EOD (Scheme 5). The radiolabelling of the amyloid-β plaque imaging agent [^11^C]PiB was achieved, as well, with an overall time of 50 minutes from EOD and in good RCY (38%, isolated, decay-corrected) but low *A*_m_ (15 GBq per μmol).^43^

**Scheme 6:**
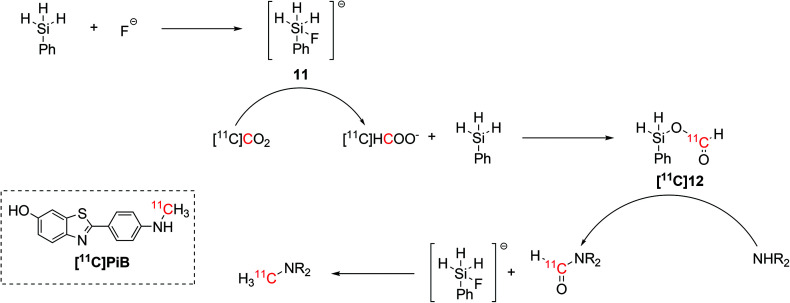
Fluoride-activated hydrosilylation of [^11^C]CO_2_ and application in the radiosynthesis of ^11^C-*N*-methylamines, including the amyloid-β plaque imaging agent [^11^C]PiB.^44^

A simpler set up that would not require the use of unstable NHCs was lately proposed for the synthesis of ^11^C-*N*-methylamines.^44^ Inspired by non-radioactive experiments,^45^ the radiolabelling step required the use of only hydrosilane and TBAF as fluoride source.^44^ The hydrosilane and the fluoride source were initially mixed in order to form an activated pentavalent fluorosilyl anion (**11**, Scheme 6) which instantly reduced the delivered [^11^C]CO_2_ to [^11^C]formate, allowing the formation of a ^11^C-silylformate ([^11^C]**12**, Scheme 6). The ^11^C-formyl group was then attached to the aminic precursor, yielding a ^11^C-formamide that was reduced to ^11^C-*N*-methylamine by the excess of fluorosilyl anion previously formed (Scheme 6).^44^

This fluoride-activated radiosynthesis of ^11^C-*N*-methylamines was also fully automated and exploited for the synthesis of [^11^C]PiB with RCY of 15% (isolated, decay-corrected) and *A*_m_ 61 GBq per μmol within 32 min from EOB.^44^

### 2.4. Organosilicon as precursors for carbon-11 radiolabelling

Recent findings revealed that organosilicon compounds are also viable radiolabelling precursors, especially in the form of trialkylsilyl and trialkoxysilyl arenes. The synthesis of these versatile organosilicon precursors is readily achieved *via* transition metal catalysis, such as Ni, Rh, Cu, allowing the silylation of a large number of aromatic compounds (*e.g.* Grignards, amides, cyanides, esters and acyl fluorides) with a wide functional group tolerance.^20,46–49^

**Scheme 7:**
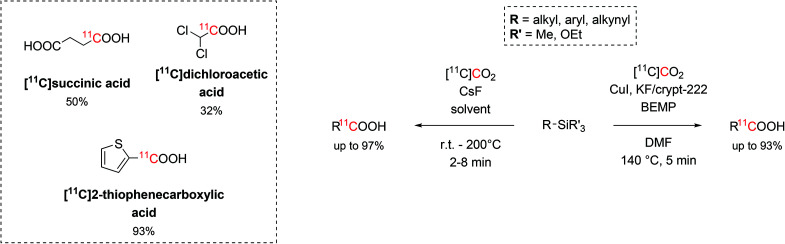
The use of trialkylsilyl and trialkoxysilyl as precursors in the ^11^C-carboxylation with [^11^C]CO_2_.

Trialkylsilyl and trialkoxysilyl arenes showed high reactivity towards copper-catalysed desilylative carboxylation reactions.^50^ With the aid of a fluoride source, the precursor was initially converted into a pentavalent silyl fluoride anion which then undergoes oxidative addition onto the Cu catalyst and react with the cyclotron-produced [^11^C]CO_2_. The highest reactivity was achieved when using DMF as solvent, KF/Kryptofix® 222 (crypt-222, 0.25 equiv.) as fluoride source, 2-tert-butylimino-2-diethylamino-1,3-dimethylperhydro-1,3,2-diazaphosphorine (BEMP, 0.6 equiv.) as CO_2_-trapping agent and a temperature of 140 °C for 5 minutes (Scheme 7). This method resulted equally efficient in the radiolabelling of alkynyl, aryl and heteroaryl precursors in short times (12 min) and with RCYs ranging between 19% and 93% (estimated by radioHPLC).^50^

A similar methodology that did not require copper catalysis was developed, as well. When CsF (1 equiv.) was employed as fluoride source and the reaction proceeded for 2–8 minutes at temperatures between r.t. and 200 °C, a large number of alkynyl, aryl and heteroaryl precursors (26 examples, Scheme 7) were successfully ^11^C-carboxylated with RCYs from 11% to 99%.^51^ A variety of solvents (DMF, DMA, DMSO/THF) were also tested returning similar trapping efficiency and reactivity. Using this strategy, eleven alkyl silyl precursors were successfully ^11^C-carboxylated (RCYs = 19%–97%), as well, including the mitochondrial kinase inhibitor dichloroacetic acid ([^11^C]dichloroacetic acid) and [^11^C]succinic acid (isolated decay-corrected RCYs = 32% and 50%, respectively, Scheme 7).^51^

**Scheme 8:**
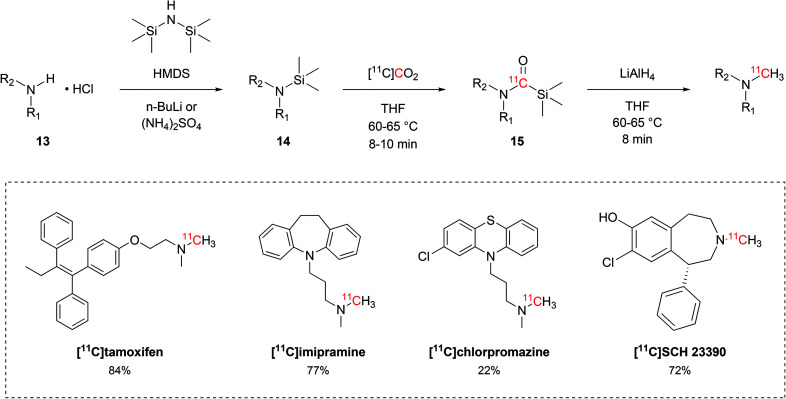
^11^C-methylation of secondary amines from [^11^C]CO_2_ and trimethylsilyl amines.^52–56^

Besides the production of ^11^C-carboxylic acids, the interaction between alkylsilyl precursors and [^11^C]CO_2_ showed to effectively yield other moieties, such as ^11^C-*N*-methylamines. Following a protocol that was initially developed with non-radioactive CO_2_,^52^ Ram *et al.* established a reliable method for ^11^C-*N*-methylation of secondary amines to methyl-^11^C-tertiary amines *via* direct use of [^11^C]CO_2_ and silyl amine precursors. The hydrochloride salt of the amine precursor (**13**, Scheme 8) was initially treated with hexamethyldisilazane (HMDS, Scheme 8) in the presence of either *n*-butyl lithium or ammonium sulphate to yield the respective silyl amine (**14**, Scheme 8). Upon delivery, [^11^C]CO_2_ would then be incorporated in the precursor as ^11^C-silyl carbamate (**15**, Scheme 8) when reacted for 8–10 minutes at 60–65 °C. **15** is then reduced by lithium aluminium hydride to the desired tertiary ^11^C-*N*-methylamine with RCYs ranging between 22% and 84% (isolated, decay-corrected at EOB) and *A*_m_ of 1.5–15 GBq per μmol (calculated at EOB) within 36–50 minutes.^52–56^ This method was applied in the radiolabelling of a variety of tertiary alkylic amines, including the biologically-active molecules [^11^C]tamoxifen,^53^ [^11^C]imipramine,^54^ [^11^C]chlorpromazine^55^ and [^11^C]SCH 23390 (Scheme 8).^56^

**Scheme 9:**
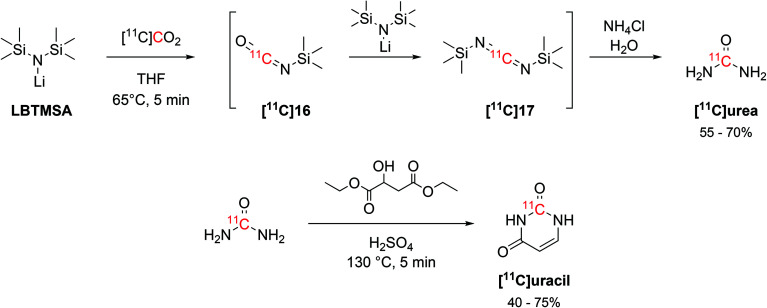
Synthesis of [^11^C]urea *via* the coupling of a silylamine precursor and [^11^C]CO_2_ and subsequent condensation with diethyl malate for [^11^C]uracil production.^57^

The interaction of alkylsilyl precursors with [^11^C]CO_2_ showed to be effective in the radiosynthesis of ^11^C-ureas, as well.^57^ The reaction uses lithium bis(trimethylsilyl)amide (LBTMSA, Scheme 9) as a precursor which reacts with [^11^C]CO_2_ for 5 minutes at 65 °C to yield the corresponding ^11^C-carbodiimide ([^11^C]**17**) species *via* a ^11^C-isocyanate intermediate ([^11^C]**16**, Scheme 9). The hydrolysis of the ^11^C-carbodiimide with an aqueous solution of ammonium chloride then produced [^11^C]urea (Scheme 9) with RCY of 55–70% (estimated by radioHPLC) in 16 minutes from EOB. The obtained [^11^C]urea was then employed in the synthesis of the nucleotide [^11^C]uracil by condensation with diethyl malate in presence of fuming sulphuric acid (Scheme 9) at 130 °C for 5 minutes. [^11^C]Uracil was obtained with RCY of 40–75% (estimated by radioHPLC).^57^

**Scheme 10:**
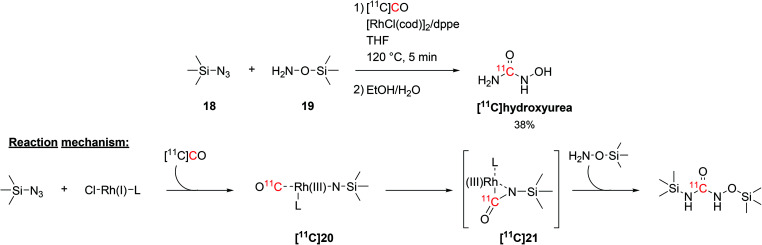
Coupling of [^11^C]CO with trimethylsilyl precursors to yield [^11^C]hydroxyurea.^58^

The radiolabelling of ^11^C-ureas could also be achieved by coupling organosilicon compounds with [^11^C]CO. This reaction requires the presence of a silylazide (**18**) and a (silyl)hydroxylamine (**19**, Scheme 10) and proceeds *via* transition metal catalysis in short times.^58^ In particular, the reaction was initially developed using trimethylsilyl azide and *O*-(trimethylsilyl)hydroxylamine in THF and in the presence of chloro(1,5-cyclooctadiene)rhodium(i) dimer ([RhCl(cod)]_2_) and 1,2-bis(diphenylphosphino)ethane (dppe) as catalyst and ligand, respectively (Scheme 10). The reaction proceeded at a temperature of 120 °C for 5 minutes and the resulting [^11^C]hydroxyurea was obtained with a RCY of 38% (isolated, decay-corrected, based on delivered [^11^C]CO).^58^ The suggested reaction mechanism proceeds with the formation of a Rh(i)-bound nitrene intermediate ([^11^C]**20**) which then reacts with the delivered [^11^C]CO to yield a Rh(iii)-coordinated ^11^C-isocyanate ([^11^C]**21**, Scheme 10). Then, the hydroxylamine acting as nucleophile reacts with the ^11^C-isocyanate forming a silyl-protected ^11^C-urea (Scheme 10). The cleavage of the silyl groups was then easily achieved with a mixture of water and ethanol.^58^

The synthesised [^11^C]hydroxyurea (Scheme 10), which in the body acts as a ribonucleoside reductase inhibitor, was then utilised to study its pharmacokinetics across the blood brain barrier *in vivo* and explore the interaction with multidrug resistance transporters.^59^ This was achieved by measuring the brain activity in rats with and without the simultaneous administration of multidrug resistance protein inhibitors (such as cyclosporin A and probenecid). The influx of [^11^C]hydroxyurea in the rat brain, however, was not significantly modified by the used intervention drugs, suggesting that hydroxyurea is not a substrate for active efflux transporters at the blood brain barrier.^59^

**Scheme 11:**
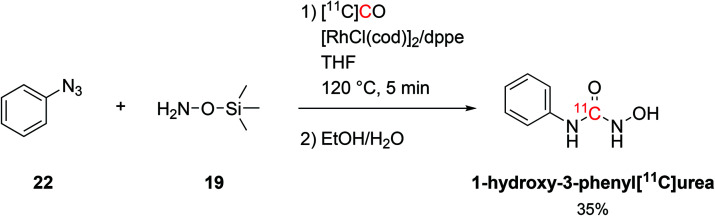
Synthesis of 1-hydroxy-3-phenyl[^11^C]urea.^58^

The coupling of organosilicon compounds with [^11^C]CO was also employed in the synthesis of 1-hydroxy-3-phenyl[^11^C]urea by using phenylazide (**19**, Scheme 11) and *O*-(trimethylsilyl)hydroxylamine as reagents (**22**, Scheme 11) whilst keeping the same conditions.^58^ 1-Hydroxy-3-phenyl[^11^C]urea was successfully radiolabelled with RCY of 35% (isolated, decay-corrected, based on delivered [^11^C]CO) and *A*_m_ of 686 GBq per μmol at 21 minutes from EOB.^58^

**Scheme 12:**
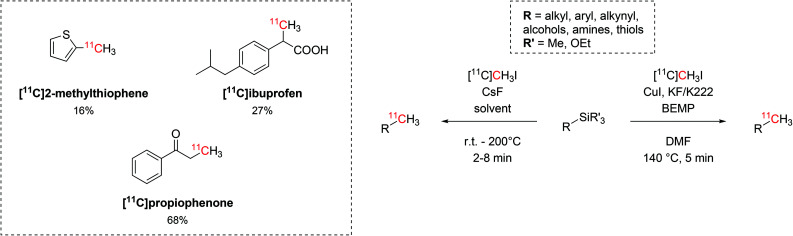
The use of trialkylsilyl and trialkoxysilyl as precursors in the ^11^C-methylation with [^11^C]CH_3_I.

The use of trialkylsilyl and trialkoxysilyl precursors was then tested for ^11^C-methylation with [^11^C]CH_3_I (Scheme 12). The radiolabelling on heteroatoms (O, N, S), aryl and alkyl precursors was readily accomplished by either copper-catalysed or fluoride-activated desilylative reactions and two biologically-active molecules such as [^11^C]propiophenone and [^11^C]ibuprofen were also produced (isolated decay-corrected RCYs = 68% and 27%, respectively, Scheme 12).^50,51^

## 3. Conclusion

In the past years, the interesting chemistry of silicon-containing compounds has been embraced for radiolabelling purposes, meeting the need for simpler, cost-effective and more efficient radiolabelling methodologies. The versatility of the silicon atom unlocked a plethora of different applications, from [^11^C]CO_2_ converting agents to radiolabelling precursors, and enabled the production of a large number of biologically-active radiopharmaceuticals. Nonetheless, the applicability of compounds bearing silicon is yet to be fully explored. Taking inspiration from conventional organic chemistry, where silicon-based chemistry is already widely utilised, many more applications could be developed for ^11^C-labelling. For example, the hydrosilylation of CO_2_ have been used for the synthesis of a larger number of chemical structures (formamides, aldehydes, aminals)^10–13^ whereas in ^11^C-chemistry it's been used only for the production of ^11^C-*N*-methylamines.^43,44^

Another potential route for widening the use of ^11^C-Si chemistry would be taking inspiration from the more established ^11^C-boron chemistry.^60^ Boron and silicon share many similarities with respect to bond energy and general chemical properties,^61,62^ suggesting that they may also have similar applicability in radiochemistry. Whilst some of these methodologies showed to be suitable for both boron- and silicon-containing molecules (*e.g.*^11^C-carboxylation with [^11^C]CO_2_, ^11^C-methylation with [^11^C]CH_3_I), organosilicates have not yet been tested in many other radiolabelling strategies, such as the ^11^C-carbonylation with [^11^C]CO or the ^11^C-cyanation with [^11^C]HCN, which instead are well-established with organoboronates.^60^ Regarding the potential coupling with [^11^C]CO, evidence from non-radioactive studies already show its feasibility: the synthesis of unsymmetrical diaryl ketones was achieved by combining a silyl arene, a iodoarene and CO in the presence of KF as activator.^63^ Likewise, non-radioactive experiments showed that the synthesis of thioesters is achievable by coupling CO with organosilicon precursors.^64^

The development of novel organosilicon radiolabelling strategies could also take inspiration from the well-established radiochemistry of organostannic compounds.^65^ Exploiting the Stille coupling reaction, the use of organostannanes as radiolabelling precursors found several applications and was coupled with a variety of carbon-11 synthons, such as [^11^C]CH_3_I, [^11^C]CO and [^11^C]acyl chloride, for the production of a variety of functional groups (*e.g.* aryl ^11^C-methylated compounds and ^11^C-ketones).^65^ The translation of these methodologies into silicon-based ^11^C-chemistry would represent a big step forward.

Besides the development of novel methodologies, organosilicon radiochemistry may also benefit from broadening the pool of radiolabel-able substrates. The previously discussed techniques are indeed still limited to certain classes of precursors, like the reductive functionalisation of [^11^C]CO_2_ (Scheme 6) which was poorly tested on alkyl precursors. The development of a “universal” radiolabelling tool, applicable to a larger number of radiotracer regardless of their chemical nature, should then also be taken into consideration.

The use of the radiochemistry of organosilicon compounds should also be enhanced in regular radiopharmaceutical production. The aforementioned methods are still restricted to the research world and not have impacted the broader clinical production of radiotracers yet. Although some clinically-used radiotracers were successfully produced with the discussed methods (*e.g.* [^11^C]PiB *via* reductive functionalisation of [^11^C]CO_2_),^43,44^ traditional radiolabelling processes (*e.g.*^11^C-methylation with [^11^C]CH_3_I) are still preferred for routine clinical production in spite of the advantages that novel methods would bring to routine production such as the much shorter radiolabelling time. When considering [^11^C]PiB labelling, for example, the traditional production *via*^11^C-methylation with [^11^C]CH_3_I requires the prior conversion of [^11^C]CO_2_ to [^11^C]CH_3_I, a time-consuming step (around 5 minutes)^66,67^ that instead is not needed in [^11^C]CO_2_-based silicon chemistry. Moreover, the presence of a free hydroxyl group on [^11^C]PiB demands for protection/deprotection^68^ to avoid side reactions whilst silicon-based methods do not have these limitations as specific towards amines.^44^ All these factors significantly lower the radiolabelling time from 15 to 1 minute (from end of [^11^C]CO_2_ delivery to end of synthesis (EOS)),^44,68^ a critical improvement considering the short half-life of ^11^C. The use of organosilicate methodologies would also lower the costs as not requiring the purchase and maintenance of costly infrastructure such as a standalone methyl iodide production unit – potentially increasing the availability of carbon-11 labelled compounds in laboratories. The RCY of the final radiopharmaceutical would also benefit from the use of the aforementioned methods (12% with [^11^C]CH_3_I *versus* 26% with silicon chemistry).^44,68^ Hence, the translation of silicon radiochemistry into PET laboratory routine production is another important step that is required.

## Conflicts of interest

There are no conflicts of interest to declare.

## Supplementary Material
